# Supplementary material for: MetaRibo-Seq measures translation in microbiomes
Source: Nat Commun. 2020 Jun 29;11:3268. doi: 10.1038/s41467-020-17081-z (PMC7324362; doi:10.1038/s41467-020-17081-z)
Supplement: Supplementary file 10 — Supplementary Data 7 [file 41467_2020_17081_MOESM10_ESM.zip › File2/Confidence_High_Taxonomy/181231_out.krona.html]

Javascript must be enabled to view this page.

members
magnitude
magnitudeUnassigned
count
unassigned
taxon
rank

181231\_out

3

2
3
superkingdom

1239
3
phylum

3
class
186801

order
3
186802

541000
family
3

genus
3
1263

1638786
3

SRS014979\_contig\_number\_contig-100\_1093.180405SRS104311\_contig\_number\_22082SRS144183\_contig\_number\_25389
species
